# Aggressive Cutaneous Squamous Cell Carcinoma of the Head and Neck: A Review

**DOI:** 10.3390/curroncol30070487

**Published:** 2023-07-11

**Authors:** Neha Desai, Mukul K. Divatia, Aniket Jadhav, Aditya Wagh

**Affiliations:** 1 JDC Healthcare, Houston, TX 77015, USA; 2 Houston Methodist Hospital, Houston, TX 77030, USA; mdivatia@houstonmethodist.org; 3 VCU School of Dentistry, Richmond, VA 23298, USA; 4 JDC Healthcare, Houston, TX 77034, USA

**Keywords:** aggressive cutaneous squamous cell carcinoma, head and neck region, review, non-melanoma cancer, metastatic

## Abstract

Non-melanoma skin cancer of the head and neck (NMSCHN) is one of the most common malignancies worldwide, and its incidence is growing at a significant rate. It has been found to be aggressive in its spread and has the capacity to metastasize to regional lymph nodes. Cutaneous squamous cell carcinoma (cSCC) has a considerably high mortality rate. It has remarkable characteristics: diameter >2 cm, depth >5 mm, high recurrence, perineural invasion, and locoregional metastases. Aggressive cSCC lesions most commonly metastasize to the parotid gland. Also, immunocompromised patients have a higher risk of developing this aggressive cancer along with the worst prognostic outcomes. It is very important to discuss and assess the risk factors, prognostic factors, and outcomes of patients with cSCC, which will give clinicians future directives for making modifications to their treatment plans. The successful treatment of aggressive cSCC of the head and neck includes early detection and diagnosis, surgery alone or adjuvant chemotherapy, and radiotherapy as required. Multimodal therapy options should be considered by clinicians for better outcomes of aggressive cSCC of the head and neck.

## 1. Introduction and Epidemiology

Non-melanoma skin cancer is the most common malignancy found across the world with a rising incidence rate from 3 to 8% annually since the 1960s. Basal Cell Carcinoma (75%) along with squamous cell carcinoma (25%) are the most important non-melanoma cancers affecting the skin. Cutaneous squamous cell carcinoma (cSCC) is the next most common carcinoma in Caucasians after basal cell carcinoma, affecting about one million people annually and contributing to 20% of all the cutaneous malignancies. cSCC usually metastasizes locally within 1 to 2 years after diagnosis. It is frequently found in the head and neck region as this area is exposed to sunlight and radiation more often than other areas of the body; thus, the initial spread is observed in the ipsilateral submandibular, sublingual, and intra-parotid lymph nodes. This particular cancer has a metastatic range of 2.3% and 5.2% after considering 5 year and longer follow-ups, respectively [1]. It has been reported that incidence rates of cSCC of the head and neck region are higher in patients with fair skin and continuous intense sun exposure [2]. Ultraviolet light B (UVB) has a higher carcinogenic potential than Ultraviolet light A (UVA), and both can cause cancer. Exposure to ultraviolet rays leads to the formation of pyrimidine dimer, which causes point mutations in the DNA, prompting tumor formation to begin [3]. Aggressive cSCC is characterized by a high recurrence rate which demands large surgical excisions, greater metastatic potential to regional lymph nodes, and a considerable mortality rate [4].

Annually, the incidence of cSCC is around 200,000 to 300,000, and around 2000 deaths occur every year in the United States (US) [5]. The incidence is 16 per 100,000 people in Central Europe, while it is 356 per 100,000 in sun-exposed White men in the southern US. It is mostly seen in men with an average age of 66 years [6]. Over 2.1 million patients are treated every year for non-melanoma skin cancer, as per the data by Medicare. The financial and economic burden of cSCC is greater than 29 billion dollars. The tumor suppressor gene p53 and the oncogenic activity in the RAS pathway are all involved in the tumor formation [7]. Studies have found that cSCC has a 3.7% metastasis rate and about a 2.1% mortality risk [8].

## 2. Risk Factors

The risk factors for aggressive cSCC include immunosuppression, continuous sun exposure associated with an outdoor occupation, Caucasian origin, male gender, changing social trends, and an age of 65 years and above. It has been observed that the current incidence is expected to increase with the growing ozone depletion, an increase in the number of those receiving organ transplants, and an aging population [9]. Certain genetic disorders are associated with high-risk cSCC. Xeroderma pigmentosum, oculocutaneous albinism, epidermodysplasia verruciformis, and dyskeratosis congenita are all linked to an increased risk of aggressive cSCC. Another genetic condition called recessive dystrophic epidermolysis bullosa, associated with aggressive or high-risk cSCC, shows the highest mortality. As discussed earlier, poorly differentiated lesions have a higher tendency to become aggressive or pose a higher risk, with an estimated 2.9-fold increase in distant metastasis. Increased risk is observed in certain histologic variants or subtypes of cSCC, such as acantholytic (adenoid), isolated cell pattern, adenosquamous, desmoplastic, and metaplastic subtypes of cSCC, especially when considering the tumor differentiation of these variants [10,11].

Oddone et al. analyzed 250 patients and observed that the important prognostic factors that are predictors of aggressive cSCC were immunosuppression, treatment, extra capsular spread, and the margin status [12]. Patients with recessive dystrophic epidermolysis bullosa, exposure to arsenic, psoralen-ultraviolet-A (PUVA) radiation, and other bullous disorders may develop aggressive cSCC with a bad prognosis [13].

Risk factors of aggressive cSCC significantly increase the probability of metastasis in patients [14,15]. Cutaneous squamous cell carcinomas, including aggressive ones, in the auricular region are at a significantly greater risk of developing metastasis, with up to almost 15% spreading majorly to the upper deep cervical and the parotid lymph nodes.

## 3. Immunosuppression

Immunocompromised patients demonstrate a greater risk of developing aggressive cSCC along with a worse prognosis than immunocompetent patients. They show twice the risk of metastasis and 13% more risk than immunocompetent patients, which depends on the type of immunodeficiency [15]. An increased incidence of aggressive and malignant cSCC of the head and neck region is observed in immunocompromised patients suffering from lymphoma or leukemia, or organ transplant recipients due to compromised cell mediated immunity [16]. When compared to the general population, the prevalence of cSCC is 65 times higher among organ transplant recipients, with the human papilloma virus being a significant risk factor [2,17]. Additionally, cSCC is recurrent, aggressive, and has a high mortality rate in individuals with small cell lymphocytic lymphoma and chronic lymphocytic leukemia [18]. In a study by Nguyen et al., it was observed that a small number of HIV (Human immune deficiency virus) patients developed aggressive cSCC, and 50% died at a 7-year follow-up [19]. A retrospective study by Southwell et al. examined the outcomes of metastatic cutaneous cSCC to the parotid and neck after management by parotidectomy and neck dissection. It was found that the recurrence rate of local and distant metastases was higher in immunocompromised patients (56%) compared to the immune competent group (28%). Also, the survival rates were significantly reduced at 1- and 2-year follow-ups. At a 2-year follow-up after treatment, only a small proportion of immunosuppressed patients were alive, as opposed to 87% of the immunocompetent group [20].

## 4. Clinical, Histological, and Pathological Features

Aggressive cutaneous squamous cell carcinoma of the head and neck (cSCCHN) can present itself clinically as an erythematous papule, plaque, or ulcer (non-healing) and is more likely seen in areas of the body that are exposed to the sun, such as the nose, ear, cheek, lips, and frontotemporal areas. These lesions can be painful or painless and bleed occasionally. Some with verrucous growth patterns can present themselves as slowly growing wart-like lesions, locally invasive, or scaly. Patients with Bowenoid papulosis display hyperpigmented papules while epidermodysplasia verruciformis patients show white flat lesions [21]. Aggressive cSCC lesions are typically characterized by a diameter > 2 cm, depth > 5 mm, high recurrence, perineural invasion, and loco regional metastasis [22,23]. Along with the factors described above, aggressive cSCCHN has shown invasion beyond the subcutaneous tissue regions and also a significant reduction in 3-year disease-specific survival rates [4]. Recent studies have identified a very high-risk cSCCHN which is characterized by desmoplasia, >4 cm in lesion size, depth of invasion > 6 mm or beyond of fat tissue, poor differentiation, and the presence of tumor cells present within the nerve sheath of a nerve lying deeper than the dermis [24]. Marjolin’s ulcer (MU) is an aggressive cutaneous malignant change seen in chronic wounds or burn scars [25].

Pathologically, cSCC develops from keratinocytes in the epidermis’ spinous layer. Atypical keratinocytes invade the dermis in the characteristic pattern. Compared to other types, aggressive cSCCHN is poorly differentiated, is most commonly spindle cell carcinoma, and occurs in the ultraviolet radiation-damaged areas of body. Vimentin and cytokeratin are observed in the immunohistochemical findings of aggressive cSCCHN. The desmoplastic cSCC variant, an aggressive form, presents itself as an invasive tumor, with poorly differentiated lesions and a poor prognosis. Aggressive cSCC has a pronounced component of stromal tissues and keratin pearls [21,23].

## 5. Lymph Node Metastases

Aggressive cSCC lesions most commonly metastasize to the parotid gland, the common lesions being in the pinna, cheek, temple, and forehead due to lymphatic drainage in the periparotid lymph nodes, deep parotid lymph nodes, and the upper cervical lymph nodes [22,26]. The rate of loco regional recurrence and the five-year disease-specific survival for patients having lymph node metastasis in the neck and parotid region is 50% [27]. Since patients with parotid metastases have greater rates of occult and clinical neck disease, the surgical treatment of the neck is the standard choice [28]. Moore et al. observed that lymphovascular invasion can significantly increase the risk of developing nodal metastasis and found that 40% of patients with positive nodes had lymphovascular invasion while only 8% had negative nodes [29].

A retrospective study by Kraus et al. observed that the preauricular region, the ear, anterior scalp, and the nose were commonly associated with regional node metastases [30]. In the United States, around 2500 people die annually due to nodal metastases that developed because of advanced cSCC [31]. Higher rates of extra capsular spread in the lymph nodes and the soft tissue deposits (about 70% of the patients) are a distinct feature of cSCC of the head and neck that can lead to distant metastases and loco regional failure along with reduced survival rates [32,33].

## 6. TNM Staging

The American Joint Committee on Cancer (AJCC) had grouped all the non-melanoma skin cancers comprising the cSCC together for cancer staging. The 7th edition of AJCC (AJCC 7) TNM staging, in contrast to the previous 6th edition staging, included high-risk criteria in the T staging, such as the thickness of tumor > 2 cm, Clark level > IV, perineural invasion, anatomic site, tumor diameter, invasion into the cranial bone, as well as the degree of histological differentiation [34,35].

AJCC7 stratified patients for the first time on nodal number, size, and laterality. However, it ignored the distinct biological dissimilarities between cutaneous SCC and mucosal SCC [36]. The 8th edition of the cancer staging manual by the AJCC (AJCC8), which was implemented in January 2017, recognized cutaneous squamous cell carcinoma of the head and neck as a separate entity and made several changes to the T category. The changes made to the T1 comprised tumors with a dimension ≥ 2 cm, T2 ≥ 2cm but < 4 cm, T3 ≥ 4 cm or minimal bone erosion or perineural invasion (PNE) or deep invasion. T4 was divided into T4a (extensive cortical or marrow invasion) and T4b (cranium-based invasion) [36]. AJCC8 also introduced ENE (Extranodal extension) for the first time in the N category. N1 comprised metastasis in a single ipsilateral lymph node ≤ 3 cm in the greatest dimension with no ENE. N2 comprised N2a (metastasis in a single ipsilateral node 3–6 cm, no ENE), N2b (metastasis in multiple ipsilateral nodes ≤ 6 cm, no ENE), and N2c (metastasis in bilateral/contralateral nodes ≤ 6 cm, no ENE). The N3 comprised N3a (metastasis in any node > 6 cm, no ENE) and N3b (metastasis in any lymph node with extra nodal extension). The M component comprises M0 with the absence of distant metastasis and is present in M1. This resulted in the upstaging of the disease in AJCC8, compared to AJCC7 [37,38].

In a study of 383 patients with metastatic cSCCHN by Liu et al., 74.6% patients were upstaged by the AJCC8. However, they analyzed that the prognostic information of AJCC8 was not useful and also stated that after ENE was incorporated in it, about 50% of the patients were classified as N3b and 88% as Stage IV. No patients in their study were restaged to the AJCC8 N3a category, as none had metastasis to just the lymph node (>6 cm), i.e., no extranodal extension. Due to the infiltrative nature of aggressive or metastatic cSCCHN, the N3a category of AJCC8 seems redundant. This first of a kind, large analytic study of AJCC8 for cSCCHN found that the AJCC8 performs poorly to establish prognostic value for patients [38].

Luk et al. described the predictive usefulness of the AJCC8 nodal staging method for patients with cSCCHN and found the upstaging of 80% of patients due to ENE inclusion. This study reported that almost 30% of diseased patients upstaged to Stage IV from III as compared to the earlier AJCC7. The disease-specific survival in patients between the N1, N2, and N3a category was not significantly different. The results showed that risk is poorly stratified through the nodal staging of the patients [39]. Similar observations were noted by Sood et al. in their retrospective analysis of AJCC8 staging for cSCCHN (with nodal metastasis). They concluded that the inclusion of ENE results in the remarkable redistribution and upstaging of the disease into TNM stage 4 and nodal classification pN2a and pN3b. However, they determined that incorporating it into the staging system has caused poor prognostic performance [40].

The clinical viewpoints of head and neck surgeons on the variables influencing AJCC8 staging-based prognostic classification were discussed by Watts et al. They brought to light that most head and neck cancer surgeons considered immunosuppression as a prime prognostic factor of cSCCHN based on the fact that a high incidence and rapid progression (aggressive) of this cancer is seen in solid organ transplant recipients. The majority of multivariate analyses have been unable to identify immunosuppression as an independent prognostic predictor, despite the fact that the AJCC8 acknowledges the significance of immunosuppression in terms of patient outcomes [41].

## 7. Diagnostic Examination and Imaging

Patients suspected of having aggressive cSCCHN should go through an examination of the lesion, lymph nodes, and fine needle aspiration. Aggressive surgical resection of the positive lymph nodes can help to control the local and regional disease. Adjuvant radiotherapy is vital in achieving high cure rates. Radiological imaging, such as computed tomography (CT), magnetic resonance imaging (MRI), positron emission tomography (PET), and ultrasound is used for detecting subclinical nodal spread [42]. The ability to detect central nodal necrosis, the invasion of the skull base, cartilage involvement, and extra capsular dissemination with CT has been beneficial. MRI is essential for finding neurotropic tumors, identifying tissue planes, and distinguishing muscle from dense connective tissue. These imaging methods aid in the treatment planning for tumors that have invaded deeper tissue, such as the parotid gland, bone, nerves, and lymph nodes [43]. Williams et al. found that only 50% of patients with histological perineural invasion of cSCC or BCC showed the involvement of the major nerves, such as the facial and the trigeminal, and an evidence of nerve involvement was seen on the CT or MRI [44].

Since there are no reliable prognostic models for aggressive cSCCHN, it has been challenging for clinicians to predict the risk of metastasis and death. The lack of controlled trials has made it difficult to develop a standard of care for its treatment. Before treatment planning, it is crucial to carefully analyze the immune condition of the patient as immunosuppression can increase the risk of metastases or recurrence. Regular follow-ups should take place every 3 to 6 months in the span of 5 years after treatment when 95% of the recurrence and metastasis is detected, during which a skin and full body examination and close examination of the tumor site and the lymph node region should be carried out [15].

## 8. Prognostic Factors

Size, anatomic site, recurrence, history of radiotherapy, immunosuppression, and perineural invasion are high-risk prognostic markers. A poor prognosis is associated with recurrent advanced stage cSCCHN, which also has a higher risk of parotid involvement, nodal metastases, and inadequate locoregional management. Five-year disease-free survival rates are fewer than 50% despite rigorous surgical resection, which includes a parotidectomy and neck dissection, followed by postoperative radiotherapy. Poorly differentiated carcinoma was shown to be an independent predictor of worse disease-free survival, and greater age was discovered to be an independent predictor of worse overall survival among the tumor variables. In patients with parotid involvement, neck involvement, or both, no difference was found in disease-free or disease-specific survival that was statistically significant. Also, patients with metastatic cSCCHN did not seem to be affected by the location of nodal involvement [45,46].

## 9. Treatment

Complex and aggressive cSCCHN require multimodality treatment [22]. The poor identification of such aggressive lesions along with a high recurrence and regional metastasis during the initial treatment is complicated further by the improper management of the regional metastasis [29]. Patients with aggressive cSCC lesions are frequently treated with substantial surgical removal followed by postoperative radiation because they are at significant risk of metastasis, recurrence, and inadequate local control [22].

The treatment of aggressive cSCCHN includes: surgery alone, surgery followed by adjuvant radiotherapy, surgery followed by adjuvant chemo radiotherapy, and epidermal growth factor receptor (EGFR) inhibition combined with chemoradiotherapy. Various studies of patients with cSCCHN with the associated outcomes are shown in Table 1.

### 9.1. Surgery Alone

The current treatment approach of aggressive cSCCHN is the complete surgical removal of the lesion with histologically clear margins [66]. Those lesions that fail to show a clear negative margin after surgery usually recur in spite of radiation. Due to the high degree of infiltration and invasion of the deeper tissues by the tumors in aggressive cSCC patients, there is a higher risk of positive margins after conventional excision. The preferred treatment for high-risk cSCC patients and tumors in cosmetically critical areas is Mohs surgery. Most of the surgical margin may be excised with this procedure, compared to fewer than 1% with standard excision. The recurrence rates after Mohs surgery are 90 to 94% for recurrent tumors and 97% for primary tumors [67]. Recurrence after surgical resection is very well predicted by factors like the involved margins of resection, two or more regional lymph nodes, and perineural invasion. Rowe et al. observed that the recurrence rates were comparatively lower for poorly differentiated tumors and those showing perineural invasion when treated by Mohs rather than the standard excision [15]. Cytokeratin immunohistochemical staining can be used along with Mohs surgery to achieve the peripheral margin followed by parotidectomy, the removal of perineural invasion, and bone removal by the head and neck surgeons for clearing the deep margins [68].

However, it is difficult to attain complete tumor clearance in cases of metastasis, parotid gland invasion, bone invasion, and intracranial extension along the major nerves by Mohs surgery. This necessitates the use of a multidisciplinary strategy in conjunction with a sentinel lymph node biopsy and preoperative imaging for the treatment of advanced stages of aggressive cSCCHN [69]. Surgical treatment modalities of aggressive cSCCHN may impair the functionality of the eyes, ears, or nose, resulting in apparent cosmetic and psychosocial effects. In these cases, a considerable amount of facial reconstruction is required. Such aggressive carcinomas ultimately affect the quality of life, resulting in further complications [4].

### 9.2. Surgery with Radiotherapy

Patients with aggressive cSCCHN lesions were frequently treated with major surgery followed by postoperative radiation because they were at significant risk of neck metastasis, cancer recurrence, and poor local control [22]. The 5-year disease survival rate is better with surgery and adjuvant radiotherapy (70–75%) than with surgery or radiotherapy alone [31].

One hundred and sixty-seven aggressive cSCCHN patients were treated with surgery or surgery with adjuvant radiotherapy, in which 59% of the metastatic nodes were located in the parotid or the cervical nodes and 41% had metastatic cervical nodes of levels I–V. It showed that patients who underwent the combined therapy of surgery along with adjuvant radiotherapy had lower rates of loco regional recurrence (20%) as compared to those with surgery alone (43%). Also, the 5-year disease-free survival rate was 73% for combined treatment, compared to 54% in surgery alone. It was concluded that surgery with adjuvant radiation therapy helped to achieve loco regional control rates of 70 to 80% after 5 years of treatment [70].

In another study, it was shown that the parotid gland was frequently included (56%) along with neck levels II (39%) and V (22%), and that nearly 76% of regional lymph node metastases occurred during the first 2 years. A modified radical neck dissection, superficial or lateral parotidectomy, and en bloc selective supraomohyoidal dissection were used to treat the majority of the regional lymph node metastases (80%), which were then followed by radiation (59%) in a number of cases. With surgery and adjuvant radiotherapy, the percentage of regional recurrences is noticeably lower than with surgery alone, hence supporting that radiotherapy is effective in reducing metastases to local lymph nodes. Surgery combined with radiation therapy to at least 60 GY improved the local control in patients with parotid metastases to 86% compared to 47% for those with radiation therapy alone [54].

### 9.3. Surgery with Adjuvant Chemo Radiotherapy

Chemotherapy has always been primarily used as palliative care in patients with aggressive cSCCHN until recently, where it has started playing a major role in various treatment approaches. The treatment intentions are palliative when there is a development of distant metastases, failed loco regional control, and surgery along with radiation can no longer prove to be of any benefit in treatment. A median survival rate of 4–6 months is generally seen after palliative care in untreated cSCC patients. Palliative chemotherapy has shown a response rate of 35–50% which can help to control disfigurement and improve the quality of life. Induction chemotherapy is the administration of a given number of chemotherapy cycles before the beginning of the definitive loco regional therapy. Its goal is to reduce the size of the tumor, hence making it a resectable one from a non-resectable size, and to treat subclinical systemic metastases [71]. The treatment of SCCHN by surgery and adjuvant radiotherapy was followed by chemotherapy treated with 13-cRA, IFN-a, and α-tocopherol (all with specific doses) for 12 months, with a dose modification. The combination of these three (cRA, IFN-a, α-tocopherol) showed promising results for its use as an adjuvant therapy in head and neck cancer patients [71].

Combination chemotherapy based on cisplatin along with methotrexate, bleomycin, doxorubicin, and 5-fluorouracil (FU) have been utilized for the treatment of advanced cSCC with wide outcomes and higher incidence adverse effects due to 5-FU. Oral 5-FU prodrug capecitabine is designed in such a way that it metabolizes to 5-FU within the pathological or tumor tissues and eventually produces lesser systemic toxicity [72]. Better results were seen in the phase 1 and phase 2 trials using capecitabine in conjunction with cisplatin or paclitaxel and radiation therapy [73]. The effectiveness of neoadjuvant gefitinib, an EGFR tyrosine kinase inhibitor (TKI), was assessed in a prospective phase II clinical trial by Lewis et al. by administering it before the conventional treatment with surgery and/or radiotherapy. With the concurrent use of gefitinib, the two-year overall survival rate was observed to be 72.1%, while the disease-specific survival rate was 72.1% and the progression-free survival rate was 63.6% [74].

### 9.4. Epidermal Growth Factor Receptor (EGFR) Inhibitors

The epidermal growth factor receptor (EGFR) is expressed in the human epidermis in the basal layers and the epidermal appendages and is overexpressed in the metastatic tumors of cSCC [75]. Its activation causes cell cycle progression, proliferation, survival, angiogenesis, and metastasis of the cancer cells [76]. The epidermal growth factor receptor (EGFR), a new biomarker, was found to be significantly associated with aggressive cSCCHN by Ch’ng et al. in New Zealand. The overexpression of the EGFR is independent of gene amplification and is markedly associated with the potential of aggressive cSCCHN to metastasize. Also, the study showed that EGFR overexpression was seen only in 47% of the metastatic disease [77]. Ch’ng witnessed the overexpression of the biomarker EGFR in a multivariate analysis of 54 patients with aggressive cSCCHN as compared to those having a primary tumor without lymph node metastases. Antibodies against EGFR may help to improve the rates of survival and loco regional recurrence in high-risk patients [78]. Cetuximab, which is one of the EGFR inhibitors, has shown good success in cases of unresectable cSCC, metastatic cSCC, and recurrent cSCC, along with its combination with celecoxib [79]. EGRF inhibitors and cepecitabine in the oral form is well tolerated by patients at a higher risk of metastasis and recurrence [80]. Further research is required to identify specific benefits from chemotherapy and define more effective treatment regimens [81].

### 9.5. T_4_ Clinical Trials

Most of the patients with advanced staged III/IV of cSCCHN display a poorer 5-year survival rate after surgery, radiation therapy, or both. The efficacy of postoperative radiation therapy and cisplatin infusion was evaluated by Bachaud et al. among stage III/IV patients with advanced cSCCHN. A dose of 50 mg of intravenous cisplatin was administered with hydration in 7 to 9 cycles along with radiotherapy. The loco regional failure rates were higher in patients who received radiotherapy (RT) alone (41%) as compared to those who received chemo radiotherapy (CRT) (23%). This phase III study also showed better overall survival and disease-free survival rates in the CRT group as compared to the RT group [82].

A phase III randomized trial was conducted in advanced cSCC stage III/IV disease to compare the results of CRT to RT alone. After the completion of all the treatment including surgery, it was found that 82% of the patients receiving RT alone and 98% patients receiving CRT consisting of fluorouracil and cisplatin intravenously were disease-free. Also, the Kaplan Meir projections showed the recurrence-free survivals were 51% vs. 62% and the distant metastasis-free survivals were 75% vs. 84% in the RT vs. CRT groups, respectively. Thus, it was concluded that CRT as compared to RT alone results in better chances of recurrence-free survival and disease-free rates in patients having resectable stage III and stage IV cSCCHN [83].

Palmer et al. studied the preliminary efficacy of radiotherapy and cetuximab as compared to radiotherapy alone in high-risk or aggressive cSCCHN patients. After a median follow-up of 30 months, they observed that the combination of radiotherapy and cetuximab was well tolerated among patients, and there was better long-term survival rate and less distant metastasis. This study was based on a small sample of patients and provided promising results for further research [84].

Hope health et al. conducted a phase 1 study to determine the effects of combined erlotinib and radiotherapy in T_4_ patients with advanced cSCCHN. The 2-year recurrence rate was 26.7%., the 2 year disease survival rate was 60% and the overall disease survival rate was 65% [85].

Lewis et al. studied the effects of neoadjuvant gefitinib in a phase II clinical trial that was administered prior to surgery and radiotherapy. For 47% of the patients who received surgery, postoperative radiation, and concomitant gefitinib treatment, the 2-year overall survival rate was 72.1%, the disease-specific survival rate was 72.1%, and the progression-free survival rate was 63.6%. Patients with aggressive cSCC lesions tolerated it well, and it had no negative effects on the outcome of the final treatment [74].

Treatment with Cemiplimab provides antibody immunotherapy for patients with aggressive cSCC (locally advanced or metastatic). This drug stimulates an anti-cancer response by blocking programmed cell death protein (PD-1). This is the first approved treatment by the food and drug administration (FDA) in the United States and the European union (EU) for patients who are not candidates for curative surgery or curative radiotherapy. Cemiplimab provides a promising therapeutic regimen for aggressive cSCC by virtue of its efficacy, clinically proven results, acceptable tolerability, and safety value [86].

Maubec et al. studied the efficacy and safety of pembrolizumab monotherapy as first-line treatment in patients with unresectable cSCC. Patients having immunohistochemically determined programmed cell death-ligand 1 (PD-L1) status in their cutaneous squamous cell carcinomas(unresectable) were administered pembrolizumab. The results of this trial showed the efficacy of pembrolizumab, its antitumor activity, and acceptable safety value. Most of the responses to this therapy occurred early. About 21% of complete responses were observed at a later stage, and it was also determined that baseline PD-L1 positivity had better efficacy [87].

Many investigational trials are being conducted currently to study the effect of modification in the already existing treatments. Currently, paclitaxel, docetaxel, cisplatin, and carboplatin-based regimens are being added as part of modification to the treatment regimens. Also, chemotherapeutic agents like gemcitabine, capecitabine, and ifosfamide are being included in the regimens. Gene therapies targeting the p53 gene whose mutation is seen in 45% to 70% of the cases are currently developed [88].

## 10. Complications

Many aggressive tumors are diagnosed with high recurrence, increased morbidity, metastasis, and death in almost 2500 cases per year. The poor identification of such aggressive lesions, along with high recurrence and regional metastasis during the initial treatment, is complicated further by the improper management of the regional metastasis [29]. Surgical treatment modalities of aggressive cSCCHN may impair the functionality of the eyes, ears, or nose, resulting in apparent cosmetic and psychosocial effects. In these cases, a considerable amount of facial reconstruction is required. Such aggressive carcinomas ultimately affect the quality of life, resulting in further complications [4].

## 11. Future Implications

Sentinel node biopsy (SLNB) and early intervention have been recently considered to improve the outcomes of the treatment through the early detection of the metastases. To determine whether a neck dissection is necessary, the nodes can be finely aspirated. For individuals who match the high-risk criteria for cSCCHN, sentinel lymph node biopsy can be used to identify lymph node involvement. Their criteria include tumor size >2 cm, the presence of the deep invasion of the malignancy, immunosuppression, location (involving the ear, labial tissues, or the nasal vestibule), growth from a scar tissue), other severe pathologic features, and faster growth. Civantos et al. found a negative prognostic value of 98% and false negative rate of 17% for the skin pathology after performing SLNB using this criterion [89]. It is cost-effective and precise in the staging of the N0 disease, but requires further investigation.

Ilmonen et al. observed in 63 patients with high-risk cSCC that (SLNB) is possible but does not offer much prognostic information. Despite the underlying tumors being high risk, only four individuals had a sentinel node that was positive [90]. In a study by Gore et al., patients with high-risk aggressive cSCCHN were evaluated with sentinel node biopsy either during the primary cutaneous tumor resection or at the time of secondary wide local excision from 2010 to 2013. Based on their results, they concluded that SLNB has a strong negative predictive value (0.98) and a low false-negative rate while treating high-risk cSCCHN. Determining the full impact of SLNB on survival outcomes will require a longer-term follow-up of this population [91].

## 12. Conclusions

The important consideration in the successful treatment of aggressive cSCCHN includes detection and diagnosis at an early stage, quick surgical resection with clearance of its margins, the staging (AJCC) of the lymph nodes, adjuvant radiation therapy and chemotherapy as required, and close and consistent follow-ups. Multimodal therapy including surgery and radiation should be considered in the treatment of aggressive carcinomas metastasizing to the neck. Adjuvant chemotherapy should be considered in cases of extra-capsular extension or in residual neck pathology. Close surveillance and examination within the first two years after treatment is recommended.

## Figures and Tables

**Table 1:** Summary of studies showing outcomes in patients with cSCCHN after various treatment modalities.

Author	Period of Study (Year)	n	Surgery Alone	Surgery + RT	Surgery + RT + CT	Disease-Specific Survival Rate (%)	Overall Survival Rate (%)	Loco Regional Recurrence (%)	Percent Distant Metastasis (%)
Hong et al. [47]	1989–1999	20	6	14	-	-	60	15	15
Moore et al. [29]	1996–2001	40	7	29	2	58.2	46.7	18	17.5
Oddone et al. [12]	1980–2005	250	28	222	-	-	78	72	<20
Veness et al. [48]	1980–2000	167	21	146	-	50	58	72	6
Hinerman et al. [49]	1969–2005	121	34	87	1.65	70	54	26	-
Babak Givi et al. [50]	1993–2007	51	11	40	-	-	30	84	-
Audet et al. [51]	1970–2001	56	7	37	-	72	53	21.5	-
Clayman et al. [52]	1996–2001	210	158	39	-	85	70	82.38	6.19
S.Ch’ng et al. [27]	1990–2004	67	28	39	-	54	44	52	6
Khurana et al. [33]	1983–1994	75	14	50	-	60	61	43	18
Ebrahimi et al. [53]	1980–2007	168	33	135	-	92	-	11	-
Andru-Chow et al. [54]	1960–2003	322	55	267	-	74	-	-	-
Palme et al. [55]	1987–1999	126	12	93	-	68	-	33	4.68
Ying et al. [56]	1982–2003	41	14	25	-	32	64	49	9.76
Barzilai et al. [57]	1994–2002	22	10	12	-	-	56	50	-
Bron et al. [58]	1988–1999	101	25	86	-	65	-	23	7
Dona et al. [59]	1983–2000	74	0	74	-	72	58	73	5
O’Brien et al. [28]	1987–1999	87	12	75	-	63	-	68	8
Chua et al. [60]	1980–1997	52	0	52	-	65	55	45	8
Jol et al. [61]	1977–1997	41	9	24	-	-	40	16	7
Gooris et al. [62]	1975–1998	44	12	32	-	-	-	9	-
del Charco et al. [63]	1966–1994	79	-	90	-	68	54	-	14
O’Bryan et al. [64]	2000–2011	22	4	11	1	-	-	-	-
Schmidt et al. [65]	1998–2011	113	13	100	-	83	80	16	7
Dean et al. [46]	1998–2007	72	24	48	-	47.2	-	65.27	14.2
